# X-ray pulse wavefront metrology using speckle tracking

**DOI:** 10.1107/S1600577515005433

**Published:** 2015-05-09

**Authors:** Sebastien Berujon, Eric Ziegler, Peter Cloetens

**Affiliations:** a European Synchrotron Radiation Facility, BP-220, F-38043 Grenoble, France

**Keywords:** metrology, phase sensing, wavefront, speckle

## Abstract

An instrument allowing the quantitative analysis of X-ray pulsed wavefronts is presented and its processing method explained. The system relies on the X-ray speckle tracking principle to accurately measure the phase gradient of the X-ray beam from which beam optical aberrations can be deduced. The key component of this instrument, a semi-transparent scintillator emitting visible light while transmitting X-rays, allows simultaneous recording of two speckle images at two different propagation distances from the X-ray source. The speckle tracking procedure for a reference-less metrology mode is described with a detailed account on the advanced processing schemes used. A method to characterize and compensate for the imaging detector distortion, whose principle is also based on speckle, is included. The presented instrument is expected to find interest at synchrotrons and at the new X-ray free-electron laser sources under development worldwide where successful exploitation of beams relies on the availability of an accurate wavefront metrology.

## Abstract 2

The theoretical description and experimental implementation of a speckle-tracking-based instrument which permits the characterisation of X-ray pulse wavefronts.

## 1. Introduction   

At-wavelength metrology is gaining in importance at X-ray large-scale facilities in order to take full advantage of the beams delivered by high-brilliance sources (Berujon, 2013 ▸; Sawhney *et al.*, 2013 ▸). While such metrology is highly desirable to optimize adaptive optics (Tyson, 2010 ▸), it finds also applications for monitoring and limiting the thermal effects on optics in full operation. Within at-wavelength metrology, the X-ray beam wavefront itself is recorded to deduce the shape and defects of the optics (Born & Wolf, 2008 ▸; Wyant & Creath, 1992 ▸). Substantial resources were dedicated during the last decade to reach this goal. Nowadays, several instruments or methods are available for measuring the phase of an X-ray beam. However, since most of them require several acquisitions per wavefront reconstruction, their use becomes confined to synchrotron sources for which the beam remains stable over a time period much longer than the total data acquisition time (Kewish *et al.*, 2010 ▸; Brady & Fienup, 2006 ▸). These metrology methods can also be sometimes tedious to implement since they can require hundreds of images and low-noise detectors. The context with X-ray free-electron laser (X-FEL) sources is quite different as X-rays are emitted in the form of pulses, each X-ray bunch presenting a wavefront slightly different from the others. The need for a wavefront metrology capable of analyzing each individual bunch makes many online synchrotron metrology techniques inapplicable to X-FEL sources.

The Hartman sensor is one of the few instruments capable of measuring beam wavefronts from shot to shot. This device has already been implemented at EUV FEL sources (Bachelard *et al.*, 2011 ▸) and hard X-ray synchrotrons (Mercere *et al.*, 2005 ▸) to evaluate the sphericity of wavefronts. Nevertheless, this instrument suffers from a couple of drawbacks: (i) a limited spatial resolution due to the minimum spacing and size of the grid probing holes, and (ii) the need for a delicate calibration to take into account and correct for the imperfections of the grid. The grating interferometer is another device used for single-pulse metrology at X-FELs (Rutishauser *et al.*, 2012 ▸; Kayser *et al.*, 2014 ▸; Fukui *et al.*, 2013 ▸). While the Hartman instrument does not need coherence due to the hole grid, the shearing interferometer is conversely taking advantage of contrast brought by interference. This interferometer, close in principle to the Hartman sensor, presents similar limitations, *i.e.* a trade-off must be made between spatial resolution and sensitivity while the grating quality may also affect the results. With both instruments the beam wavefront gradient is derived from a method based on the principle of wavefront modulation, the local propagation direction of the X-rays being inferred from the distortion of a reference pattern. This latter is generated either by the probing grid of holes in the case of the Hartmann or by the interferometer phase grating.

**Figure 1:**
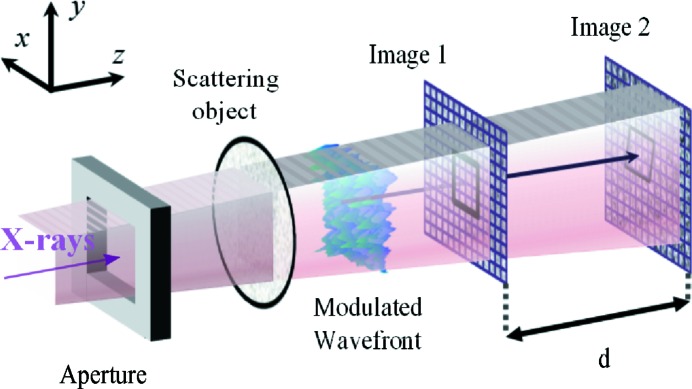
Speckle tracking principle in the absolute configuration.

Herein, we propose an original device based on the X-ray speckle tracking (XST) principle (Berujon *et al.*, 2012 ▸; Morgan *et al.*, 2012 ▸) to enable the recovery of an X-ray wavefront from two images acquired simultaneously. Previous work on XST (Berujon *et al.*, 2012 ▸) showed the way the X-ray beam absolute wavefront could be obtained through the recording of images of a scattering object at different propagation distances from the source (see Fig. 1 ▸). Now, in addition to a scattering membrane, the wavefront sensor instrument integrates a semi-transparent scintillator, a key component able to emit visible light whilst being partially transparent to X-rays. In this manuscript, special care is given to the calibration and information that can be mathematically derived. For instance, it describes the way the detector distortion is taken into account and corrected for and the way the speckle tracking implementation is enhanced. The instrument concept was experimentally demonstrated at synchrotrons. In principle, the transition to an X-FEL installation is quite straightforward providing technological issues related to the acquisition of simultaneous images can be solved. However, the instrument configuration as presented in the following is limited to the sensing of the wavefront when no sample is inserted into the beam path. Modifications to the second detector of our current instrument would be necessary to obtain a configuration where the beam wavefront is characterized before it is impinged by a sample.

## 2. Instrument presentation   

The XST principle is recalled in Fig. 1 ▸, where an X-ray beam passes through a thin phase object with random scattering grains and is then recorded at two different planes upon propagation. Due to the partial coherence of the X-ray beam, the waves scattered from the random phase object interfere with the transmitted light to generate speckle, *i.e.* random contrast features (Goodman, 2006 ▸). As the distortion of this interference pattern in the near field region is solely dependent on the wavefront propagation (Cerbino *et al.*, 2008 ▸; Gatti *et al.*, 2008 ▸; Magatti *et al.*, 2009 ▸), it is possible to deduce the wavefront state by numerical processing of the images (Berujon *et al.*, 2012 ▸).

The instrument presented in this paper employs two detectors collecting indirectly the visible light emitted through X-ray illumination of scintillators placed in series at two different propagation planes. Such a setup is comparable with the one used for instance by Carnibella *et al.* (2012 ▸). In it, the first scintillator traversed by the photons presents the particularity of being made of a thin low-absorbing material that lets most of the beam pass through it. The visible light generated by luminescence is then imaged on the CCD chip through a microscope objective and a carbon glass mirror orientated at 45° with respect to the probed beam direction and transparent to X-rays. The second camera is of a more traditional conception with a thick scintillator coupled to a microscope objective system to fully absorb the X-ray beam. The two cameras are triggered to acquire images of the beam simultaneously at the two different propagation planes.

## 3. Method principle   

### 3.1. Image acquisition and preprocessing   

The XST method relies on the availability of high-spatial-resolution hard X-ray imaging detectors. With the current technology presented above, it is hence usual during experiments to deal with noise due to, among other causes, the electronics, scintillator defects or from stray light.

Pre-processing operation can, however, strongly reduce their effects and optimize the signal-to-noise ratio of the imaging system. For instance, to compensate for some of the electronic noise, a darkfield correction can be applied to minimize the background noise. For this, many images are acquired with the detector shutter closed, averaged to a matrix 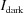 and subtracted from the images recorded with the beam on.

The non-homogeneous response of the scintillator can also be compensated through flatfield correction; an average image is generated from many acquisitions taken at various positions of the membrane across the beam. This flatfield 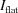 image is then used for the normalization of the image recorded intensity.

**Figure 2:**
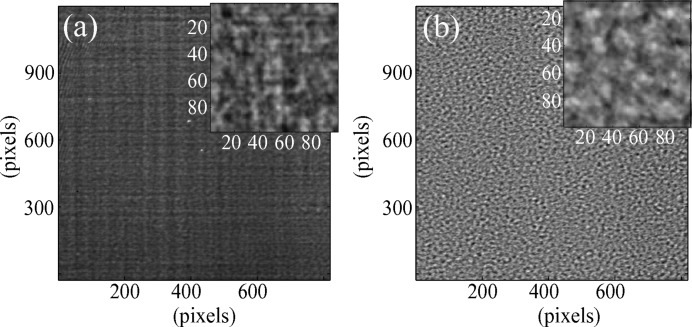
Sample image from the first detectors before (*a*) and after (*b*) normalization.

Therefore, the recorded image 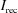 undergoes the correction
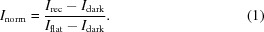
While not mandatory, this type of correction, largely used among the X-ray imaging community, was proven to avoid obvious artefacts from the scintillator structure and from defective pixels. Fig. 2 ▸ shows an example of an image before and after the correction has been applied.

### 3.2. Speckle subset tracking   

The XST principle implemented in the ‘absolute mode’ configuration (Berujon *et al.*, 2012 ▸) corresponds to the sketch in Fig. 1 ▸: the two images of the scattering object contain high-frequency features which are essentially an almost similar speckle pattern. The speckle grains are used as markers, whose shapes upon propagation can be thought of as needles, to infer the trajectory of the rays from one image to the next. This concept is realised by the search and identification of small subsets of pixels from the first image into the second one using a cross-correlation algorithm. The localization of the correlation peak provides displacement vectors 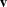 between the images for the 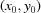 centered subsets. Noting the distance between the images *d*, the X-ray beam wavefront gradient is linked to 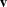 through the equation of propagation in a homogeneous media by (Berujon *et al.*, 2012 ▸; Born & Wolf, 2008 ▸)
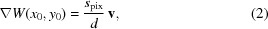
where 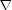 is the del operator and 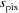 is the detector effective pixel size.

**Figure 3:**
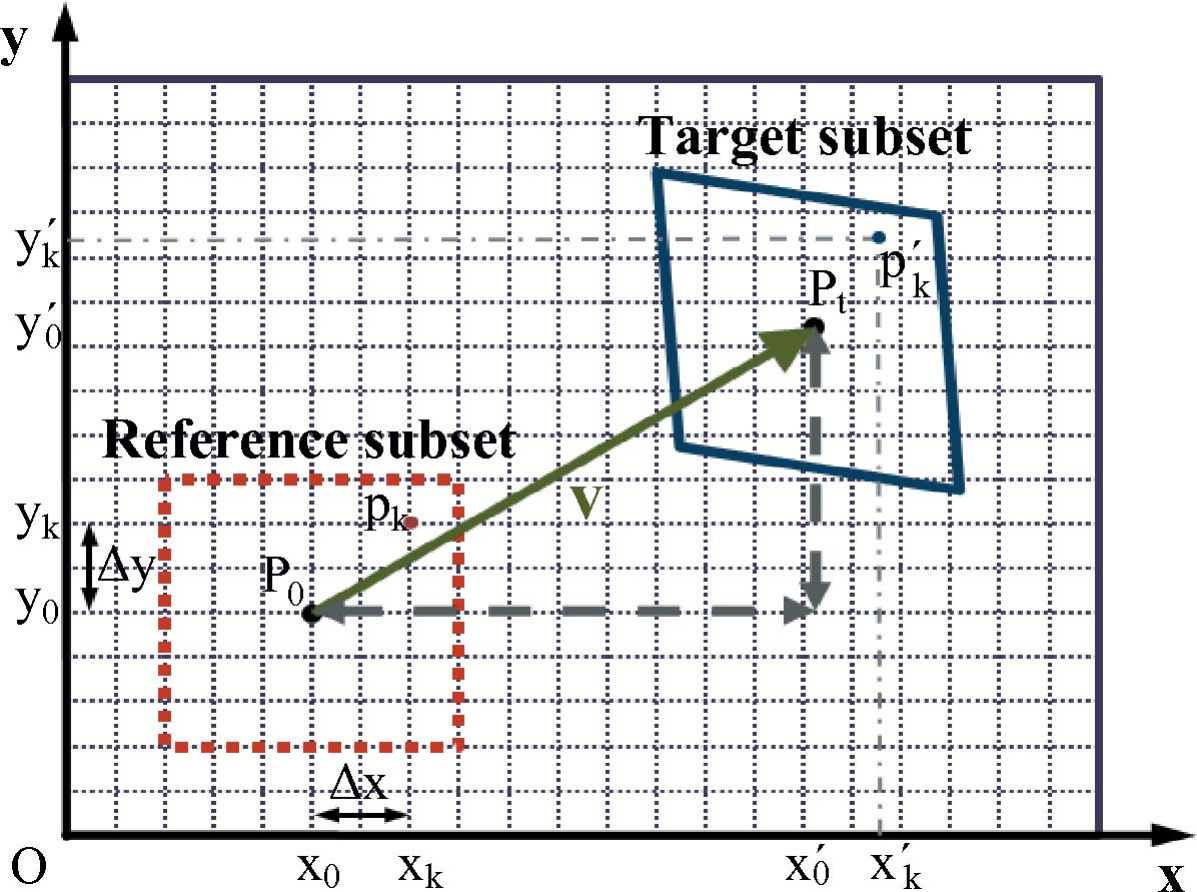
Subset tracking geometry.

The numerical foundation of the XST technique relies on the ability to track the subsets between images accurately. The geometry and notations of the subset are displayed in Fig. 3 ▸. When considering a small subset of pixels initially centered around 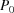 = 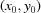 in the first image, its transformation upon propagation to the second detector and now located around 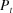 = 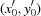 can be described by (Pan *et al.*, 2009 ▸)
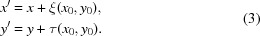
In this set of equations, the ξ and τ functions mirror both the displacement and distortion of the subset. For this subset noted *f* in the first image, 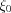 and 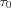 are recovered by searching the subset matching similar counterpart *g* in the second image such that 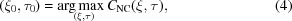
where 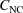 is the zero-normalized cross-correlation criterion defining the similarity between two considered subsets by
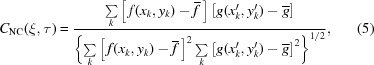
where
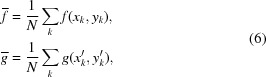
and 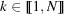, *N* being the number of elements in the subset. The subset size is usually chosen in the range 13 × 13 pixels to 27 × 27 pixels which are sizes offering a good compromise between precision, resolution and speed of calculation.

Instead of the zero-normalized cross-correlation criterion of equation (5), another equivalent criterion sometimes employed for subset tracking is the zero-normalized sum of squared difference (Pan *et al.*, 2009 ▸; Yoshizawa, 2009 ▸; Zanette *et al.*, 2014 ▸).

### 3.3. Rigid subset translation   

As described in equation (2), the recovery of the wavefront gradient is possible by simply calculating the pixel subset displacements between the images (Berujon *et al.*, 2012 ▸). Such an approach considers only
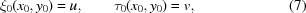
where *u* and *v* are scalars. Using a so-called peak finder algorithm, the localization of the maximum correlation between the subset and the target images provides the coordinates of the displacement vector 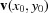 = 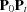 = 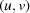 in the basis transverse to the beam. The precision of the tracking algorithms can be easily improved to reach a fraction of a pixel by fitting the correlation peak neighbor pixels to a Gaussian or polynomial surface (Pan *et al.*, 2009 ▸).

### 3.4. Subset distortion   

As pointed out in previous work (Berujon, 2013 ▸), the advantage of considering the subsets distortion is twofold: (i) it makes the tracking algorithm more robust and accurate, and (ii) it brings additional information on the local higher wavefront derivative orders. For instance, the local curvature is accessible through the calculation of the second-order subset distortion setting: 
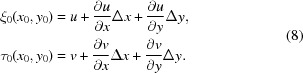
In this set of equations, 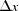 = 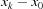 and 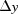 = 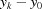 are the distances from the subset center *P*_0_ to a point of the subset *p*_*k*_ (see Fig. 3 ▸), and 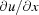, 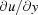, 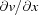 and 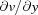 are the first-order distortion factors of the 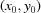 centered subset.

These coefficients can be calculated by Newton-based minimization of the functional
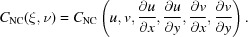
Such an optimization algorithm has a radius of convergence of the order of a pixel, implying the need for a first calculation step to extract the pixel accurate rigid subset motion. To achieve a subpixel accuracy within this kind of algorithm, the second image is interpolated and the correlation coefficient 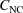 calculated using an updated target subset defined by ξ and τ (Bruck *et al*., 1989 ▸; Vendroux & Knauss, 1998 ▸).

The coefficients 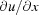 and 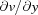 correspond to the magnification 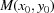 = 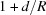 between the reference and the matching target subset. Optically, the local wavefront curvatures in the detector basis is hence
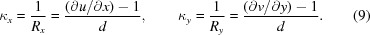
Similarly, it can be shown that the cross terms 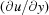 and 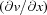 are linked to the subset rigid body rotation 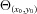 by
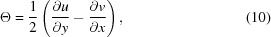
which is valid for small angles (Vendroux & Knauss, 1998 ▸).

## 4. Detector characterization and distortion correction   

**Figure 4:**
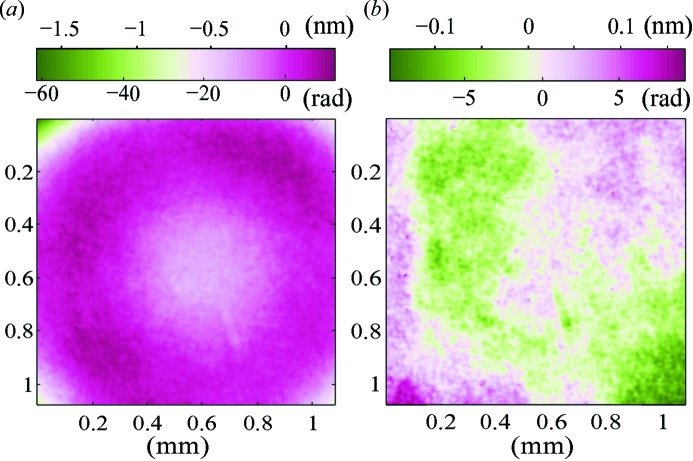
Wavefront error calculated without (*a*) and with (*b*) correction for the detector distortion.

As the optics used within the imaging detector to obtain a high resolving power is not aberration free, the recorded images become distorted. Fig. 4 ▸ shows, for instance, the strong contribution of the detector in the measurement of the wavefront error when the distortion is not corrected for.

One common way of retrieving the optical distortion of an imaging system is to image a reference grid with well known characteristics and compare it with the expected pattern. Such a calibration is effective in imaging experiments since it concurrently corrects for the distortions induced by both the visible microscope objective and the X-ray beam aberration. Here, because the X-ray wavefront aberration is the object of investigation, we only aim at correcting the detector distortion.

Unlike in methods usually employed for lens correction, we did not make any assumption on the distortion or use a distortion model for modal reconstruction (Brown, 1971 ▸), employing instead a zonal reconstruction. The proposed approach is based on a principle of rigid-body speckle translation, similar in some aspects to the method described by Yoneyama *et al.* (2006 ▸), which consists of imaging a static speckle pattern whilst moving the detector transversally in the beam propagation direction.

Let us write the real coordinates of an ideal distortion-free image point 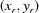 as a function of its distorted counterpart 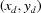: 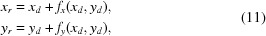
where 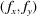 = 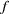 is a pair of functions describing the amounts of distortion in the detector basis 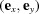.

**Figure 5:**
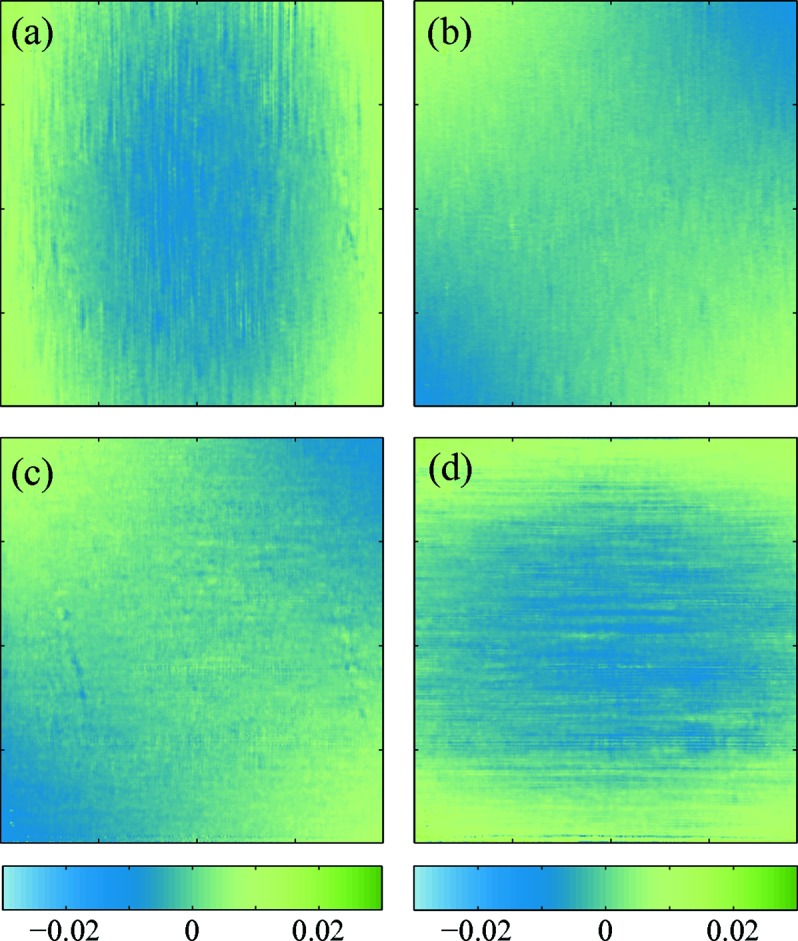
Directional derivative components of 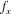 and 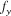: (*a*) 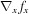, (*b*) 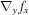, (*c*) 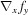 and (*d*) 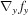. The scale is dimensionless as it corresponds to a variation of pixel size per pixel.

**Figure 6:**
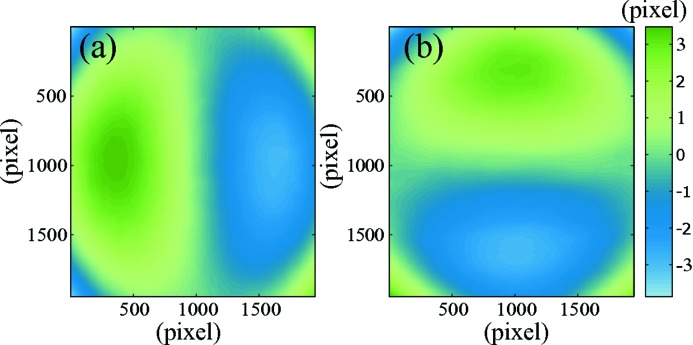
Detector distortion in the (*a*) 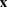 and (*b*) 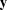 direction.

Hence, when translating the detector by the amount 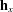 = 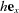 and tracking the speckle subsets between the images, the calculated displacement vectors are equal to
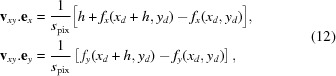
and, equivalently, when applying a displacement 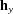 = 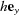,
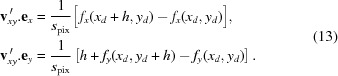
Since we can set the average of the function 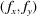 to 0 (no image translation), the effective detector pixel size 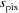 can be taken as 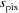 = 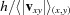 where 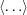 denotes the mean operator. Moreover, from the equation sets (12) and (13), we can write the approximate directional derivatives of 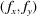: 
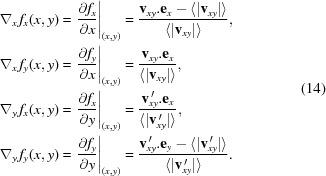
The calculated functions for our first detector are shown in Fig. 5 ▸. As mentioned previously, whilst some authors calculated and used only two of these maps to recover the lens distortion parameters of a standard model (Yoneyama *et al.*, 2006 ▸; Pan *et al.*, 2013 ▸), we use instead all of the gradients maps to reconstruct the distortion functions. The next step is hence to numerically integrate the pairs of gradient maps 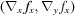 and 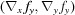. This is done by matrix inversion to find the intermediate function 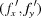 by least-square minimization of the functionals (Harker & O’Leary, 2008 ▸),
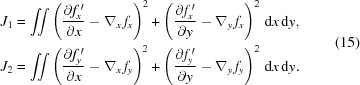
Finally the integration constants are set so that 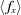 = 0, 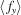 = 0, *i.e.* 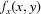 = 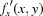 − 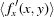 and 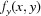 = 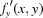 − 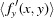. The functions 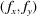, displayed in Fig. 6 ▸, were used to undistort the recorded image from the detector by bi-cubic interpolation.

The approximate directional derivatives in equation (14) are obtained by forward difference. In practice, to increase the precision of the method, we used the central difference (Riley *et al.*, 2006 ▸). Noting 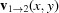 the displacement vector calculated taking the reference subset in image 1 and target subset in image 2 and 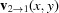 its reciprocal obtained searching the subset from image 2 into image 1, we derived the central vector value 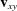 = (1/2)[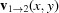 − 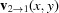].

The proposed method permits us to isolate the detector distortion and correct the images for it, thus leaving the X-ray beam aberration untouched. Correcting for the optical aberrations of both the beam and the detector, as it can be of interest for imaging purposes, would require scanning the position of the scattering object rather than the detector position.

## 5. Experiment   

**Figure 7:**
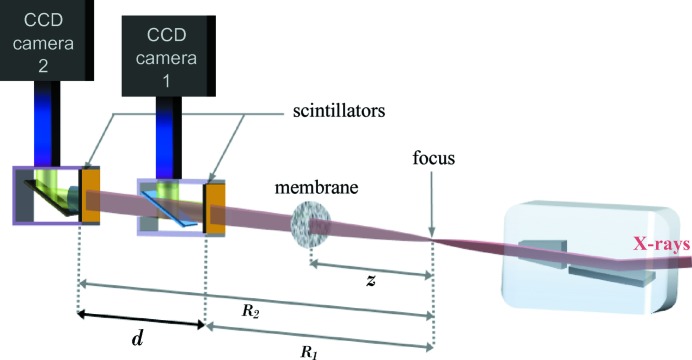
Setup of the experiment. The key element of the system, represented in light blue, is a carbon glass mirror reflecting the visible light emitted by the scintillator and letting the X-rays pass through. The distances 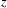 = 285 mm and 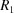 = 885 mm were fixed, whereas the distance *d* was first set to 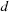 = 255 mm and later to 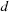 = 510 mm.

The experimental setup sketched in Fig. 7 ▸ was tested at the former ESRF beamline ID22-NI on the 6 GeV storage ring of the ESRF (Martinez-Criado *et al.*, 2012 ▸). An undulator was tuned to produce a peak of photons at an energy of 29 keV. At a distance of 64 m from the source, a Kirkpatrick–Baez (KB) bender system focused the beam by the combination of a 180 mm focal length vertical focusing mirror and a 83 mm focal length horizontal focusing mirror (Barrett *et al.*, 2011 ▸). The combined energy resolution of the undulator harmonic with the multilayer coating on the KB mirrors provided a total energy selectivity of ∼1.5%. The aperture of the KB system was defined by a pair of slits opened to 260 µm vertically and 160 µm horizontally. The size of the source was defined in the vertical direction to ∼20 µm by the synchrotron source and in the horizontal direction 25 µm by a pair of slits generating a virtual source. With such a source and KB optics configuration, a focused beam of ∼50 nm × 50 nm could be expected.

A biological filter made of cellulose acetate with a nominal pore size of 0.45 µm was placed at 285 mm downstream of the mirror focus. Both detectors composing the system were CCD-based FReLoN cameras (Fast REad out LOw Noise), relying on indirect illumination of a scintillator through Olympus visible-light microscope optics. Their equivalent pixel sizes were 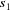 = 0.681 µm and 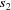 = 0.756 µm for the upstream and downstream detectors, respectively. The two scintillators were produced by liquid phase epitaxy; the first one had a 26 µm-thick crystalline layer of LuAG:Ce as active material and the scintillator of the second detector used a 24 µm-thick layer of LSO:Tb. The first scintillator had a theoretical absorption of about 23% and the second one of ∼30%. The higher absorption and then efficiency of the second scintillator was used to compensate for the beam divergence and then lower photon flux at the second detector position. The first detector was mounted on a stage fixed along the 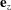 axis at 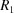 ≃ 885 mm from the focal plane of the KB optics and motorized to move within the 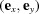 plane, while the second detector could be translated along the beam axis 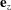. Data recorded at two detector interdistances, 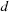 = 255 mm and 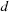 = 510 mm, allowed us to compare these two metrology data sets between them and to compare the single-pulse metrology with that employing only one detector. In the latter approach, the wavefront is derived from the analysis of two images recorded sequentially by the same (second) moving detector (Berujon *et al.*, 2012 ▸).

Dark images and flat images were generated for both detectors from image stacks as explained and shown in §3.1. The alignment of the two detectors was made so that both of their fields of view intercept the central part of the expanding beam. However, a fine adjustment is not necessary since an offset between the alignment axis of the two detectors would translate in the measurement of an additional optical tilt component. Such optical aberration is not a proper wavefront error and can be easily removed numerically.

### 5.1. Effect of the X-ray transparent mirror and scintillator   

**Figure 8:**
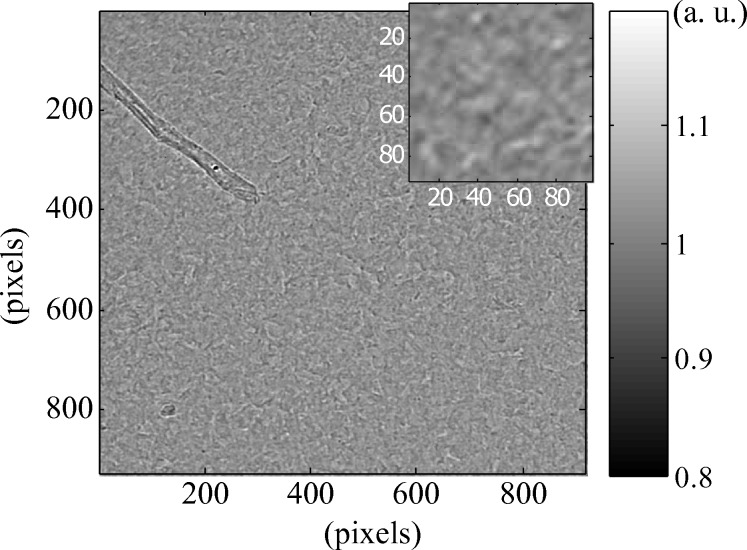
Effect of the semi-transparent mirror and first detector scintillator on the X-ray beam as seen by the second detector.

The effect of the X-ray transparent mirror and first detector scintillator on the X-ray beam was first observed. Fig. 8 ▸ shows their combined effect on the images as recorded by the second detector after a normalization. As one can see, micro phase contrast features are observable over the full field of view. Judging by their small size and relative low contrast, they are most likely due to inhomogeneities caused by the scintillator and the polishing of mirror substrates.

Images acquired during a scan of the first detector across the beam whilst the second one was kept static were used to estimate the phase effect of the transparent detector on the X-ray beam. Speckle tracking was performed between the images taken with the second detector. The displacement vectors calculated across the field of view showed that the phase effect of the X-ray transparent mirror and scintillator is negligible on the low- and mid-spatial frequencies of the beam wavefront. Indeed, the displacement vector field was shown to be 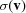 > 0.03 pixel over the full field of view, corresponding to the noise level expected for the method (Berujon *et al.*, 2012 ▸). This corroborates the conclusion that optical windows and transmission homogeneous objects generate very little phase shift as compared with reflective optics, although tending to affect the beam coherence through bulk scattering as observed in Fig. 8 ▸ (Berujon, 2013 ▸).

### 5.2. Wavefront sensing and reconstruction   

After detector calibration, pairs of images were acquired simultaneously with the two detectors. Images from each detector were then applied appropriate normalization and distortion correction. Square subsets of size 21 × 21 pixels and centered on each pixel of the first image were searched across the image from the second detectors.

At a distance 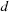 = 255 mm, the expected magnification of the speckle pattern from one detector to the other was 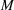 ≃ 1.16 considering setup geometry and detector pixel size difference, and of 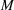 ≃ 1.42 for a distance 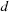 = 510 mm. Upon propagation with magnification factors close to unity, as in our case when 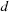 = 255 mm, the numerical algorithm can easily deal with the corresponding insignificant speckle pattern distortion upon propagation. Conversely, higher magnification factors and hence larger pattern distortions can compromise the cross-correlation calculation of equation (5), affecting therefore the ability of the algorithm to match subsets between the images. To overcome this issue encountered for instance when 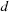 = 510 mm, the second image was undersampled by cubic bilinear interpolation in order to obtain 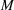 ≃ 1 after processing.

The vectors 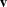 were hence calculated for each pixel of each set of images. As the pixel size differed for the two detectors, the wavefront gradient calculation had to account for this distinctive feature.

If we consider the case 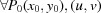 = (0, 0), we have the theoretical magnification 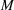 = 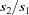 and radius of curvature 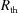 = 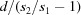, which, in our particular case, is equal to 2.315 m. Considering the linear contribution of the small angles, the calculated wavefront gradients had to be adjusted by the offset 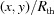,
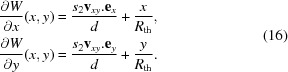
Or, equivalently, one can use a corrected displacement vector 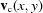 obtained using the definition of 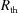,
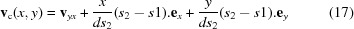
and 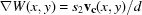.

**Figure 9:**
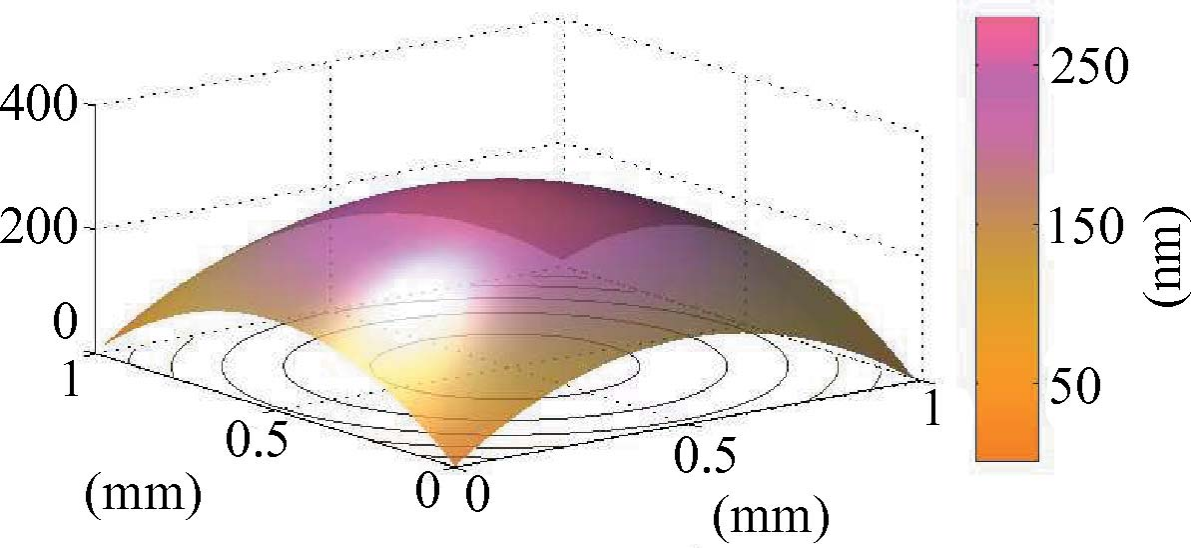
Wavefront reconstruction of the X-ray beam at the propagation plane 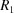 from the mirror focus.

The wavefront recovery was performed by two-dimensional numerical integration of the phase gradient maps using the numerical recipe described by Harker & O’Leary (2008 ▸) based on least-square minimization. An example of wavefront surface reconstruction at the plane 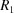 is shown in Fig. 9 ▸ for a millimeter square aperture. The wavefront radii of curvature were calculated by fitting the wavefront gradients to linear planes or alternatively the wavefront to an ellipsoid. The extracted values, 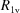 = 886.8 mm and 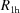 = 887.3 mm, are consistent with the hand-measured values.

## 6. Results and analysis   

### 6.1. Wavefront and wavefront gradient error   

The wavefront error was calculated by subtracting the best ellipsoid from the reconstructed wavefront and similarly the wavefront gradient error was obtained by removal of the best-fitted plane.

**Figure 10:**
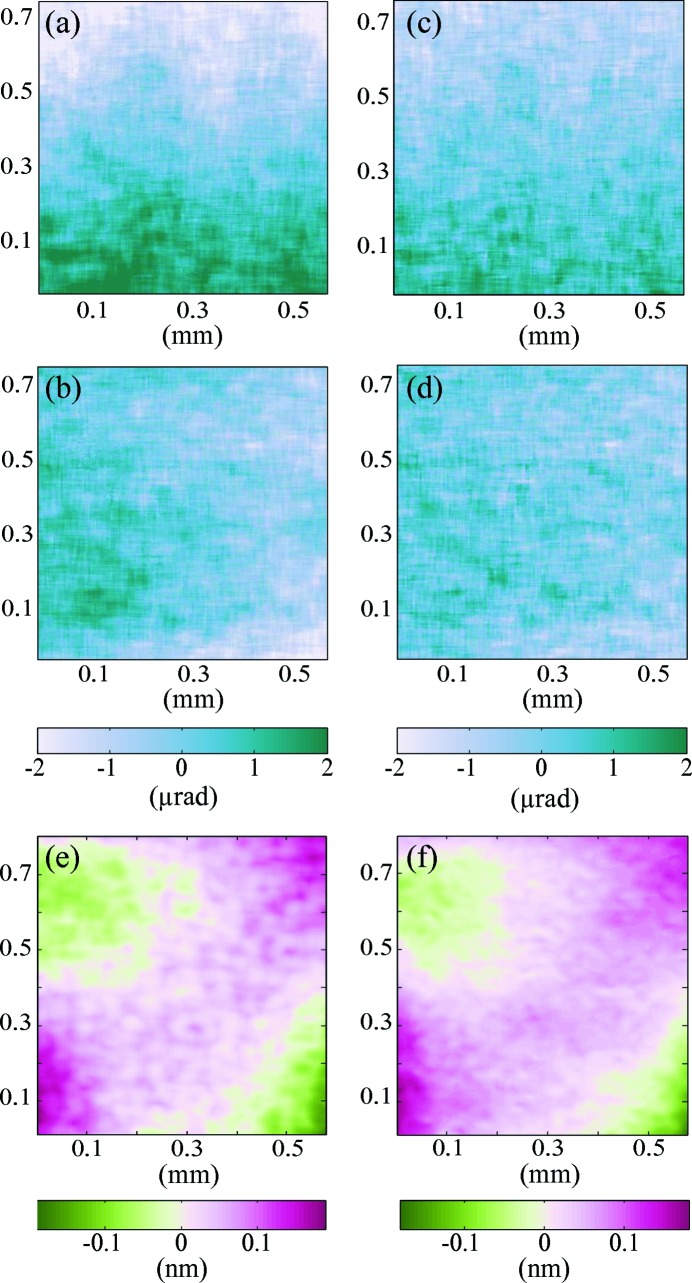
Horizontal (*a*) and vertical (*b*) wavefront gradient error maps measured for 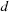 = 255 mm. (*c*, *d*) Similar measurements at 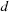 = 510 mm. (*e*, *f*) Corresponding wavefront errors of the beam.

The wavefront gradient errors and wavefront error of the measured beam are displayed in Fig. 10 ▸ for detector interdistances of 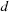 = 255 mm and 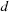 = 510 mm. Therein, the field of view displayed in the figure corresponds to the common beam area intercepted by the second detector at these two positions *d*. As a matter of fact, as the beam divergence beyond the focal plane is relatively important, moving the second detector further downstream from the focus reduces the ratio of the beam imaged with respect to the total transverse beam area.

The first observation from Fig. 10 ▸ is that the metrology measurements performed at two detector interdistances are in good agreement as we find features of comparable shape and amplitude. The main error components on the wavefront gradients are observable orthogonally to the gradient directions. This effect, equivalent to a 45° astigmatism (Wyant & Creath, 1992 ▸), may be due to small sagittal focusing effects or orthogonal misalignment of the two mirrors composing the KB optical system. We note that the pencil beam technique widely employed to optimize mirror focusing is unable to observe such an effect since the slope measured is averaged along the mirrors’ tangential focusing length. This aberration is also seen in the wavefront error shown in Figs. 10(*e*) and 10(*f*) ▸. The standard deviation of the wavefront gradients are of 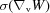 = 0.70 µrad vertically and 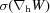 = 0.43 µrad horizontally. However, the sensitivity of the system was not optimal due to the fixed position of the first detector. When going further away from the focal point, the wavefront gradient amplitude diminishes as the radius of curvature becomes larger, making our approach less sensitive to the variation of the involved deflection angles.

### 6.2. Subset distortion calculation   

**Figure 11:**
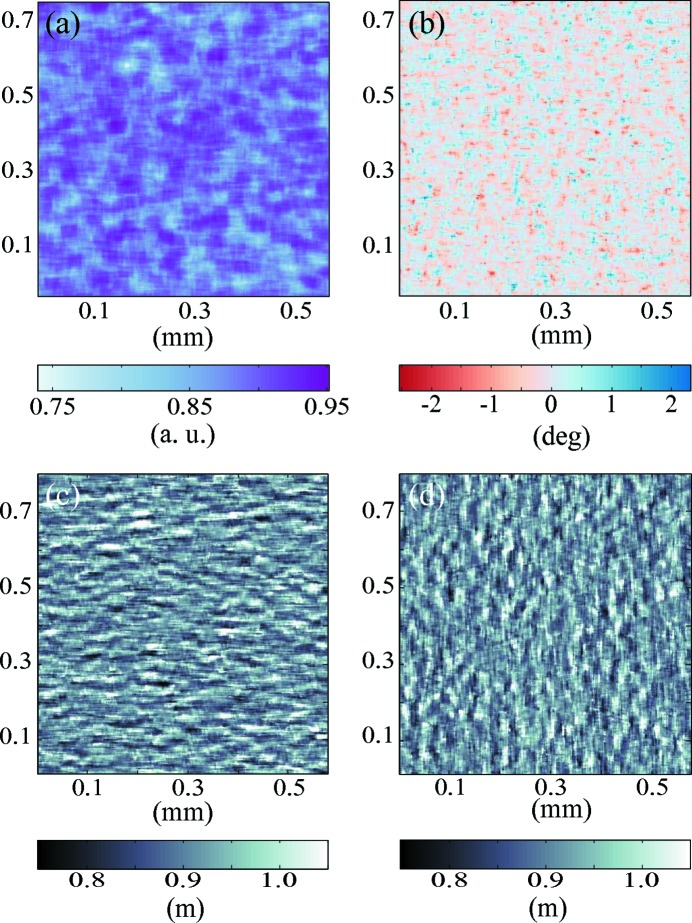
(*a*) Correlation factor between reference and target subsets. (*b*) Rigid subset rotation in degrees. (*c*) Vertical and (*d*) horizontal local radii of curvature.

The subset distortion was calculated following the method explained in §3.4. A first calculation step of the displacement vector field was performed considering only the rigid translation. Later the subset distortion algorithm was applied, the outcome of the first step being used as an initial guess for the Newton minimization algorithm. The interpolation of the target subset performed with a factor of 100 provides an expected accuracy for the displacement vector better than 0.03 pixel (Yoshizawa, 2009 ▸). Fig. 11 ▸ shows in (*a*) the correlation factor calculated at each position, in (*b*) a display of the subset’s rigid body rotation and in (*c*) and (*d*) the local vertical and horizontal radii of curvature, respectively. Considering our metrology instrument setup and the quality of the optics employed, the local radii of curvature and small rotation are in good agreement with the experimental geometry and quality of the optics.

### 6.3. Sensitivity and robustness   

The correlation factor 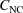 was on average ∼0.70 when considering only the rigid subset motion by peak finding algorithm and ∼0.89 when taking into account the subset distortion as described in §3.4. Despite not being a precise criterion for accuracy, this factor demonstrates that the calculation of the first-order subset distortion improves noticeably the robustness of the method. As a comment, in digital image correlation, the sharpness of the correlation peak and the peak to average correlation value are sometimes employed as reliability markers of the calculation quality. Yet, this factor can be also affected by diverse parameters such as the size and sharpness of the speckle grains. In the presented instrument, the characteristic features of the X-ray transparent mirror need to be carefully defined so as not to degrade the beam coherence or to affect the speckle pattern.

We investigated the minimum counting statistics required by the algorithm to render accurate metrology information. A series of acquisitions were performed while decreasing the exposure time on the two detectors until reaching the failure of the method. Images with counts as low as 200 (SNR ≃ 2) were successfully processed by the cross-correlation algorithm, demonstrating a good robustness to Poisson noise.

The device sensitivity relates to the smallest measurable angle at the location of the first detector scintillator. It can be estimated with 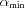 = 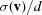 where the measurement accuracy on the vector 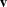 is a function of the cross-correlation algorithm, the image noise and the speckle pattern quality. Previous works showed that its accuracy could be easily pushed to <0.03 pixel (Pan *et al.*, 2009 ▸). Naturally, the instrument sensitivity is also inversely proportional to the distance *d*. Therefore, a large propagation distance is expected to increase the device sensitivity, while, in practice, this gain is moderated by the beam partial coherence and relative magnification between the images, thus limiting the maximum propagating distance usable.

## 7. Conclusion   

An instrument was developed to analyze quantitatively the wavefront of a beam from a single pulse without the need of a previous reference. The presented method offers high spatial resolution and sensitivity on the wavefront gradient measurement: whilst the resolution is of a few pixels, the angular sensitivity can be easily pushed down to <100 nrad. Moreover, we presented a way of characterizing and correcting for the distortion when performing speckle tracking with imaging detectors and we gave insights on means of developing this technique principle further. Thus, despite requiring significant computing resources, the integration of the subset distortion in the processing was demonstrated as an efficient way to boost the robustness and capabilities of the method. For the clarity of the demonstration, this paper was purposely limited to the measurement of typical incident wavefronts in the absence of a sample in the beam. However, several modifications of the instrument can be envisaged to allow the presence of a sample, for instance for imaging that would benefit from the presence of a wavefront sensor. The main modification will consist of fitting the second detector with a semi-transparent scintillator. The proposed instrument will open new opportunities in the exploration of the wavefront of X-ray pulses provided by the new X-FEL sources.
